# The Function of cGAS-STING Pathway in Treatment of Pancreatic Cancer

**DOI:** 10.3389/fimmu.2021.781032

**Published:** 2021-11-09

**Authors:** Ghazal Mohseni, Juan Li, Abakundana Nsenga Ariston Gabriel, Lutao Du, Yun-shan Wang, Chuanxin Wang

**Affiliations:** ^1^ Department of Clinical Laboratory, The Second Hospital, Cheeloo College of Medicine, Shandong University, Jinan, China; ^2^ Department of Clinical Laboratory Diagnostics, School of Medicine, Cheeloo College of Medicine, Shandong University, Jinan, China

**Keywords:** pancreatic cancer, cGAS-STING pathway, immunotherapy, type I interferon (IFN), cytosolic DNA

## Abstract

The activation of stimulator of interferon genes (STING) signalling pathway has been suggested to promote the immune responses against malignancy. STING is activated in response to the detection of cytosolic DNA and can induce type I interferons and link innate immunity with the adaptive immune system. Due to accretive evidence demonstrating that the STING pathway regulates the immune cells of the tumor microenvironment (TME), STING as a cancer biotherapy has attracted considerable attention. Pancreatic cancer, with a highly immunosuppressive TME, remains fatal cancer. STING has been applied to the treatment of pancreatic cancer through distinct strategies. This review reveals the role of STING signalling on pancreatic tumors and other diseases related to the pancreas. We then discuss new advances of STING in either monotherapy or combination methods for pancreatic cancer immunotherapy.

## Introduction

Pancreatic cancer remains an exceedingly fatal malignancy and is anticipated to become the second cause of cancer death in the USA. The survival rate during diagnosis is 10% in the USA (1). Family history, obesity, type 2 diabetes, and tobacco use are the high-risk factors for pancreatic cancer. Patients are typically diagnosed with advanced disease levels due to a lack of symptoms during the early stages (2). It has recently been reported that pancreatic cancer can be linked to many infections. There is an escalation of risk in patients with Helicobacter pylori (H-pylori) or hepatitis C infections (3, 4). Four vital mutated genes significantly distinguish pancreatic cancer. The most crucial altered gene within cancer comprises K-ras, the proto-oncogene, which is found active in its mutated form above 90% of the cases (5). However, the tumor suppressors are also modified, such as CDKN2A (6), p53 (7), and DPC4/SMAD4 (8). The treatment process of pancreatic cancer is complicated because this disease is extremely dangerous. Most patients are diagnosed late; also, pancreatic cancer has a special TME that requires more beneficial targeted therapies (9). The pancreatic tumors avoid immune responses through different strategies. Firstly, the pancreatic TME has a large variety of immunosuppressive cells such as myeloid-derived suppressor cells (MDSCs), regulatory T cells (Tregs), tumor-associated macrophages (TAMs), and immunosuppressive antigen-presenting cells (APCs) (10–13). Secondly, the leukocytes, which promote metastasis, are the important component of pancreatic tumors (14, 15). Additionally, surgery and chemotherapy treatments have poor survival results for pancreatic cancer patients (16). However, immunotherapy has recently been shown to be another important anti-tumor method in the treatment of pancreatic cancer because it induces long-lasting responses and prevents recurrence through long-term memory function of the adaptive immune system (17, 18) also by targeting and modulating the immune system, it can increase the sensitivity of cancer cells to chemotherapy. Many studies have been conducted and focused on different immunotherapy strategies in pancreatic cancer, such as immune checkpoint inhibitors, natural killer cells and dendritic cells, targeting myeloid cells and tumor-associated macrophages (19, 20). Another interesting application of immunotherapy in pancreatic cancer is the activation of type I interferons (IFNs). Type I IFNs, all of which bind to a cell surface receptor complex (IFNAR), are anti-tumor cytokines and the regulators for innate immunity activation (21). Downregulation of IFNAR1 (one of the chains in the receptor complex) lets tumor elude the IFN pathway which causes cancer development (22). It has also been reported that inactivation of the IFN1-IFNAR1 pathway by cancer-associated fibroblasts (CAFs) results in stromagenesis and growth of tumors in the colon and pancreatic cancer (23). Besides, to deal with the role of IFN I in pancreatic cancer, another research showed that type I interferons such as IFNα and IFNβ have radiosensitizing effects in pancreatic cancer, which can enhance the responses to treatment (24). Also, another research introduced an important function of IFNβ in growth inhibition in pancreatic cancer even at low concentrations (25). Hence it is worth studying pathways that stimulate the production of type I IFNs. To date, multiple stimulators for IFN I have been identified, among which stimulator of interferon genes (STING) represent a crucial one. STING resides in the endoplasmic reticulum (ER). It initiates phosphorylation and activation of the transcription factor IRF3 (interferon regulatory factor 3), which can enter the nucleus to promote the transcription of inflammatory genes, such as IFNβ (26). This pathway influences the pancreas by modulating T cell production so that when STING is activated in human cells, it can decrease the infiltration of T cells (27). In recent years, cGAS-STING signalling has become a popular mechanism to improve the immune system against malignancy. In this article, we describe the effect of cGAS-STING signalling on the pancreas. We further show the recent advances of this pathway in pancreatic cancer treatment.

## The Basic Outline of cGAS-STING Pathway

**Figure 1:**
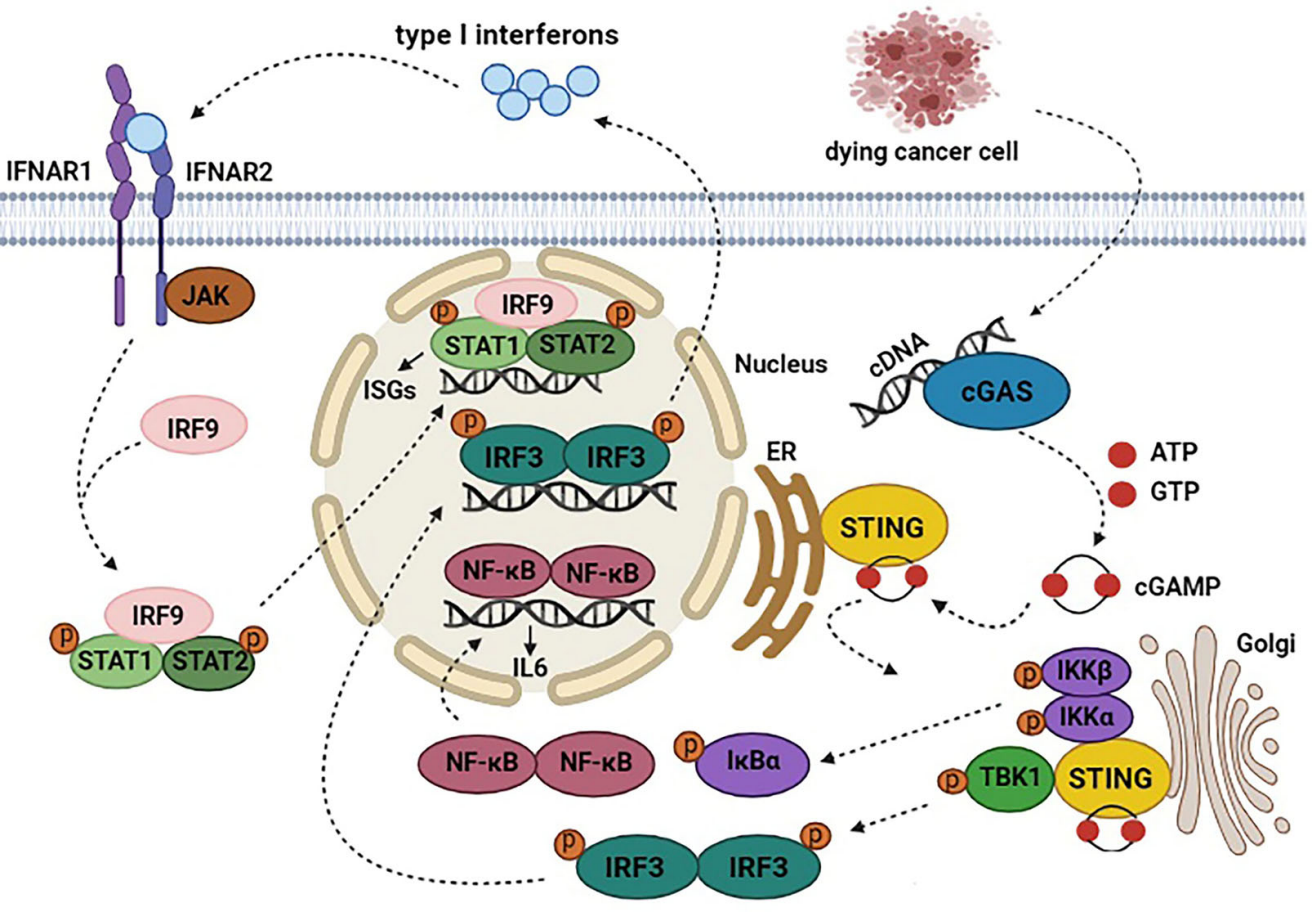
Illustrates the cGAS- STING pathway. The cyclic guanosine monophosphate-adenosine monophosphate (cGAMP) synthase (cGAS) functions as an adjuvant for stimulator of interferon genes (STING). cGAS detects the cytosolic DNA and then binds to STING and activates it. The activated STING is translocated to Golgi and stimulates the production of type I IFNs and NF-κB through tank-binding kinase 1 (TBK1)/interferon regulatory factor 3 (IRF3). The type I IFNs binds to the Interferon-α/β receptor (IFNAR) and activates Janus kinase (JAK)- signal transducer and activator of transcription proteins (STAT) pathway. The phosphorylated STAT1 and STAT2 join interferon regulatory factor 9 (IRF9) and make the interferon-stimulated gene factor 3 (ISGF3) complex, which increases the expression of IFN-stimulated genes.

The Innate Immunity is activated through recognition of the different pathogens, which depends on pattern recognition receptors (PRRs), and pathogen-associated molecular patterns (PAMPs), which are the ligands of PRRs (28). Cytosolic DNA (cDNA), an important PAMP over infection, makes DNA sensors to prompt downstream of innate immunity (29). Innate immunity performs an important function in recognizing cDNA by activating a special pathway called cGAS-STING (30). This pathway acts as the detector of self-DNA released from tumor cells and dying cells (31). The destruction of cellular homeostasis will result in the accumulation of DNA in the cytoplasm, which can bind to the cGAS and result in its activation. The cGAS remodels adenosine 5′-triphosphate (ATP) and guanosine 5′-triphosphate (GTP) to activate cyclic GMP–AMP (cGAMP). Subsequently, cGAMP acts as a messenger and transmits the signal to the downstream endoplasmic reticulum (ER) protein named stimulator of interferon genes (STING also known as MITA [mediator of IRF3 activation], ERIS [endoplasmic reticulum IFN stimulator], MPYS [N-terminal methionine–proline-tyrosine–serine plasma membrane tetraspanner], or TMEM173 [transmembrane Protein 173]). When the activated STING is translocated to Golgi, it triggers the essential signals through tank-binding kinase 1 (TBK1)/interferon regulatory factor 3 (IRF3) for production of type I IFNs and NF-κB (through IKKα/β cascades). Next, IRF3 and NF-κB target the nucleus and increase the infiltration of type I IFNs and Interleukin 6 (32–34). The type I IFNs binds to the IFNAR1/2, heterodimeric receptor, which initiates Janus kinase (JAK)- signal transducer and activator of transcription proteins (STAT) pathway (35). The JAK kinases phosphorylate STAT1, and STAT2 and interferon regulatory factor 9 (IRF9) joins STAT1/2 to make the interferon-stimulated gene factor 3 (ISGF3) complex. This complex functions as a transcriptional factor and prompts the expression of IFN-stimulated genes (ISGs) (36) **Figure 1**. All the details mentioned above indicate that cGAS functions as an adjuvant for STING. The activated cGAS binds to STING, which triggers the phosphorylation of IRF3. Following the cytosolic DNA, the cGAS-STING pathway induces immune responses (37).

## cGAS-Sting pathway and Pancreas

**Figure 2:**
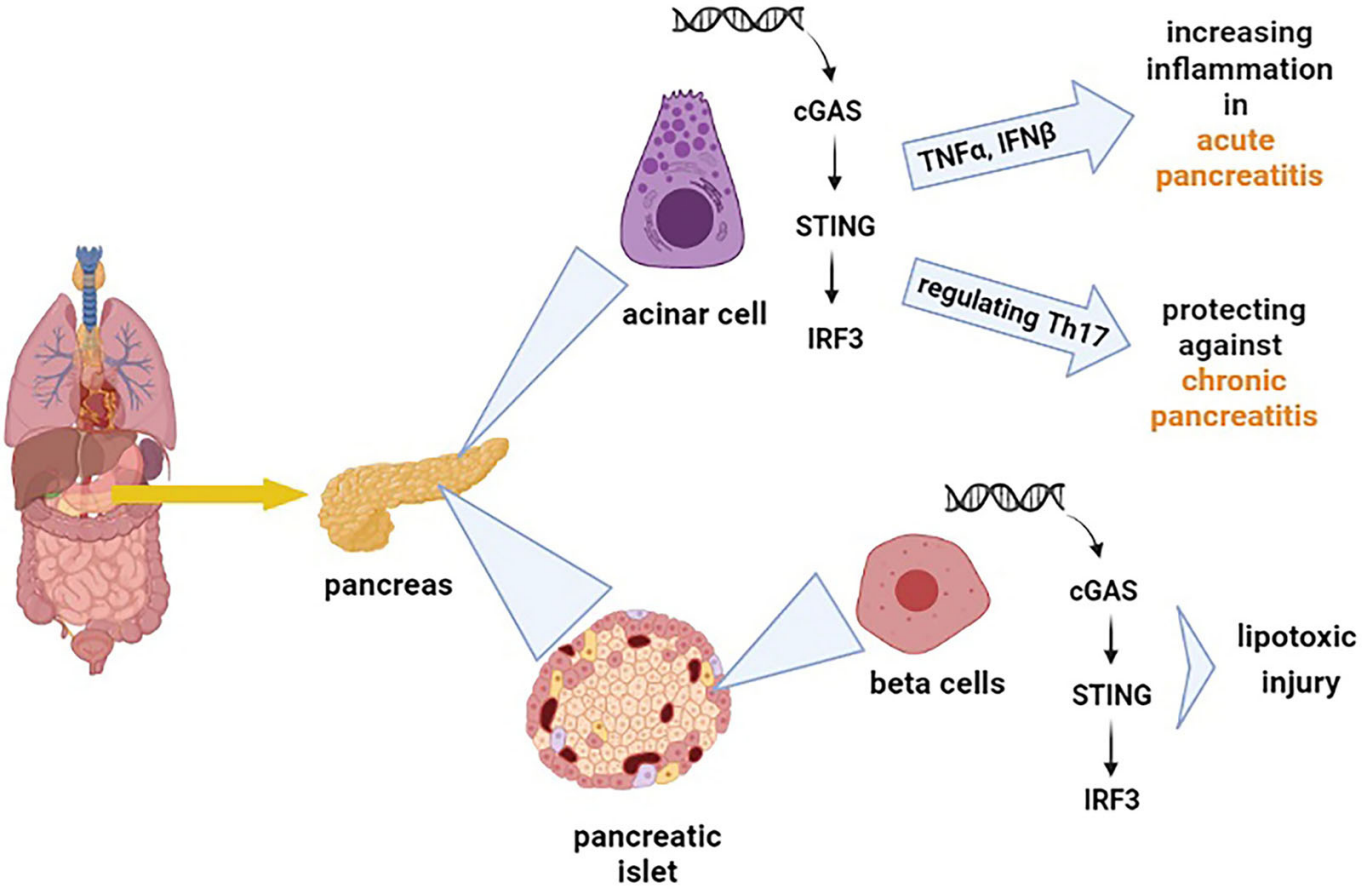
Shows the effect of the cGAS-STING pathway on different types of cells in the pancreas. The cGAS-STING pathway exacerbates acute pancreatitis by increasing tumor necrosis factor-alpha (TNFα) and IFNβ. On the other hand, this pathway regulates the production of Th17, which protects against chronic pancreatitis. The cGAS-STING pathway is also involved in the lipotoxic injury of β cells.

The cGAS-STING signalling pathway can release the type I IFNs and inflammatory cytokines, affecting the immune responses against different diseases variously (38). Inflammation of the pancreas, known as pancreatitis, is a common digestive disease, and if it develops quickly, it can cause severe damages (39). The cGAS-STING pathway has been discovered to have opposing effects on different types of pancreatitis (acute pancreatitis and chronic pancreatitis). Zhao et al. demonstrated that the STING pathway exacerbates acute pancreatitis by increasing TNFα and IFNβ (40). However, this pathway has a protecting impact on chronic pancreatitis by modulating the infiltration of Th17 (41). As chronic pancreatitis increases the risk of pancreatic cancer (42), exploring the role of STING signalling in pancreatitis provides insights into the application of STING for the diagnosis and treatment of pancreatic cancer. Pancreatic islets also function as an endocrine gland which means that it secretes hormones such as insulin and glucagon. The pancreatic islets include different cells that among them β cells are responsible for releasing insulin (43). Interestingly, it has also been found that STING is hugely expressed in mouse and human islet β cells (44), and the STING pathway has been reported to be implicated in even some islet β-cell damages (lipotoxic injury of β cells) (45). The lipotoxic injury of pancreatic β cells is one of the major hallmarks of type 2 diabetes (46). Moreover, the STING pathway has been suggested to cause suppression of diabetogenic T cells (47), which means that STING can impact diabetes and influence the integration and secretion of insulin. Based on the facts mentioned above, it is understandable that the cGAS-STING pathway has a wide and diverse range of effects on the pancreas **Figure 2**.

## STING and Pancreatic Cancer

The relationship between DNA damage and cancer has been well studied. There are multiple networks in a cell that respond to DNA damage. There are two aspects in the link between cancer and possible outcomes of immunotherapy. Firstly, DNA damage can improve anti-tumor immunity in both natural immune reactions and treatment procedures. Secondly, genome instability is a hallmark and a driving force of cancer (48). STING is a vital component for promoting tissue repair pathways which also responses to intestinal damages because DNA damage induces STING activity leading to cytokine production. These results can indicate the possible role of the STING pathway in preventing tumorigenesis (49). The cGAMP activates the STING signal pathway, which promotes the formation of IFN-γ from CD8+ T cells to reduce MDSCs and delay their immune-suppressive activities (50). Moreover, STING can have an important anti-tumor impact on TME by producing type I IFN and priming T cells *via* CD8α+ DCs. In addition, Batf3-lineage DCs respond to IFN-β produced downstream of STING growth and can present the antigens to CD8+ T cells (51). STING can be activated by tumor DNA which produces type I IFN through the STING-IRF3 axis, or cytosolic DNA activates the cGAS-STING pathway and type I IFN production, independently of APC phagocytosis (52). Thus far, a piece of research showed that the cGAS–STING pathway, activated in APCs by cytosolic DNA, provides an important source of type I IFN signalling, and it is essential for checkpoint blockade and anti-PD1 therapies (53). However, current trials are still trying to confirm the effectiveness of combination STING/anti-PD1/PD-L1 approaches. Some other studies reported the correlation between defective STING pathway activity and cancer incidence (54, 55). For example, a recent study revealed that the downregulation of the STING pathway could cause cancer resistance to immune effectors because downregulated STING pathway causes reduction of intratumoral CD8+ T cell infiltration (56). Furthermore, cancer cells can survive and evade immune responses through harboring deficiencies in the cGAS-STING pathway (57). IFN plays an important role in tumor-specific T cell responses which means that the cGAS-STING pathway is a crucial mechanism to drive inflammation-driven tumor growth. It has been reported that STING signaling plays an important role in regulating immune cell infiltration in the TME (58). These findings prove that cGAS-STING signalling is an important pathway for anti-tumor responses and immunotherapy purposes. On the other hand, Pancreatic cancer is an example of a solid tumor that evades the immune system’s surveillance. As mentioned earlier, cGAS-STING signalling can control the immune responses against different pancreatic diseases. Besides, many recent studies have been carried out to find the relationship between this pathway and pancreatic cancer so that it can be applied for pancreatic cancer therapy aims. In this part, we want to focus on the current achievements of the STING pathway in the diagnosis and treatment of pancreatic cancer.

## STING Affects Pancreatic Tumors Through Different Strategies

### STING and DNA Damage

The cGAS-STING pathway acts as a cDNA detector that activates the immune responses against cancer cells. This ability of the STING pathway has sparked developments in cancer immunotherapy. For example, the inhibition of Ataxia telangiectasia mutated (ATM) protein stimulates the cGAS-STING pathway. ATM is an essential kinase for repairing DNA double-stranded breaks. Importantly, the deficiencies in ATM can release the mitochondrial DNA into the cytoplasm, which initiates the STING pathway and infiltration of T cells (59). Recent work has shown that deficiency of ATM and activation of cGAS-STING pathway improve the immune checkpoint blockade responses in pancreatic cancer by stimulating type I IFN (60).

### STING and Cell Death

**Figure 3:**
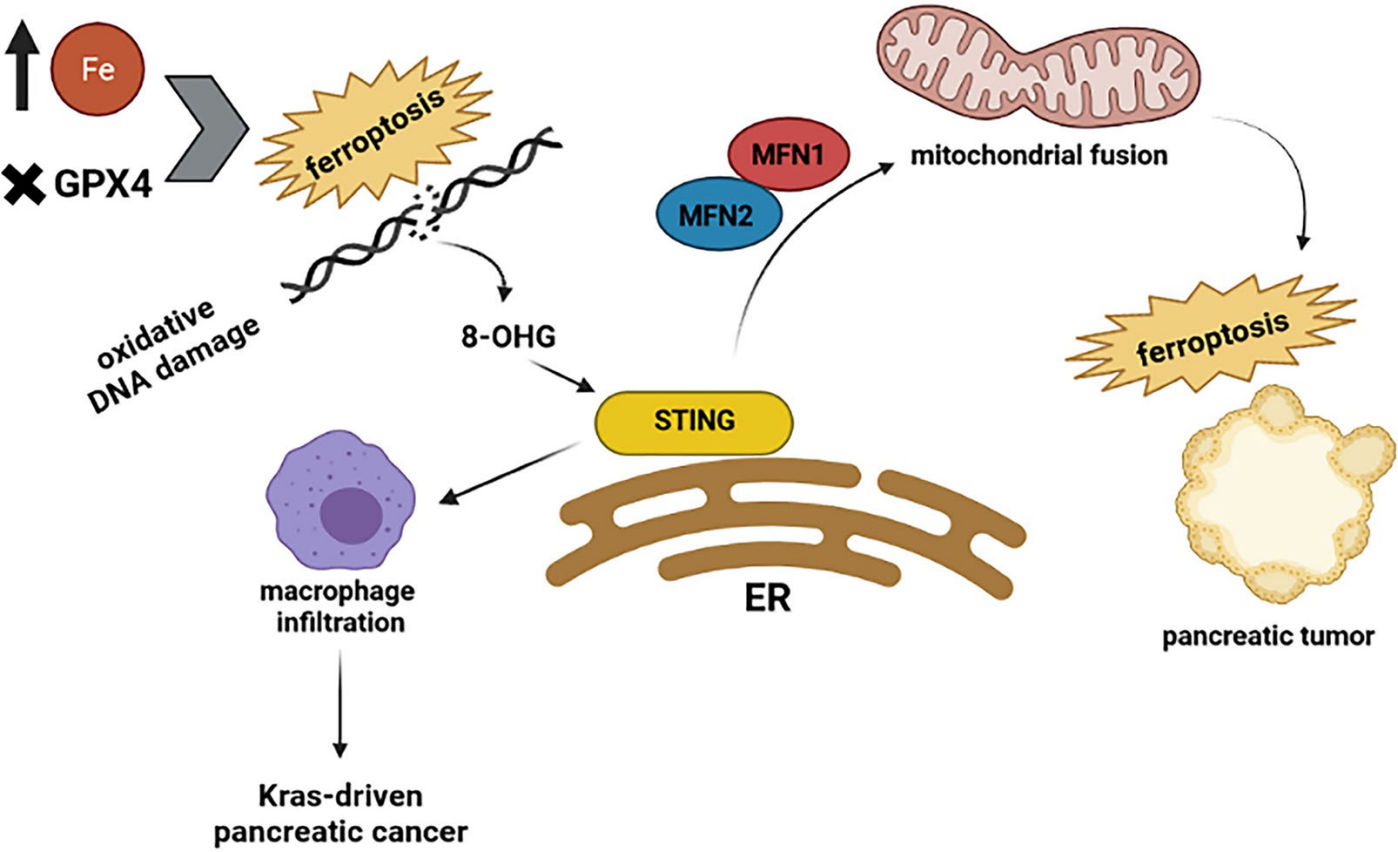
Highlights the relationship between STING and ferroptosis. The excessive amount of iron and Gpx4 reduction can induce ferroptosis and production of 8-OHG, which leads to activation of the STING pathway and infiltration of macrophages during Kras-driven pancreatic cancer. However, when STING binds to mitofusins (MFN1/2) activates mitochondrial fusion, which increases the ferroptosis in pancreatic cancer cells.

The cGAS–STING pathway can connect DNA damage to anti-tumor responses such as cell death and immune surveillance. Constant stimulation of the cGAS–STING pathway leads to cell death, promoting resistance to tumorigenesis (61–63). To support the interaction between the cGAS-STING pathway and cell death, several pieces of evidence prove this pathway causes different types of cell death such as necroptosis (64), apoptosis (65), pyroptosis, and ferroptosis (63). The early stages of different cancers are developed from faulty cell death (66, 67). For instance: activation of the STING pathway prompts necroptosis in colon cancer cells by increasing the expression of RIPK3 and MLKL, which are crucial proteins for the necroptosis process (68). Furthermore, STING has been reported to interact with spleen tyrosine kinase (Syk) and regulate pyroptosis in colitis-associated colorectal cancer (69). In small cell lung cancer, DNA damage activates the STING pathway and consequently stimulates the expression of PD-L1 and related apoptotic responses (65). Recent analysis reveals that ferroptosis, a regulated cell death, depends on iron and is specified by the accumulation of oxidative damage. Induction of ferroptosis is not just dependent on those mutations involved in the Ras pathway; it means that it can occur in a Ras-independent manner as well (70–72). Ferroptosis has been found in pancreatic tumors (73) and is associated with autophagy (74). As it is presented in **Figure 3**, ferroptosis interacts with STING and affects pancreatic cancer. For example, a recent study revealed that STING promotes mitochondrial fusion-induced ferroptosis. STING can bind to mitofusins (MFN1/2) and activate mitochondrial fusion, increasing ferroptosis in pancreatic cancer cells (75). However, the interplay between STING and ferroptosis can cause aggravation of pancreatic cancer. Glutathione peroxidase 4 (GPX4), an important antioxidant enzyme, removes oxidative damage to membrane lipids and protects against ferroptosis (76). Dai E and his colleagues demonstrated that oxidative DNA damage induces Kras-driven cancers through the infiltration of macrophages. They found that Gpx4 deficiency or excessive iron accumulation causes more production of 8-OHG (an oxidized nucleobase), then it activates macrophages and produces cytokines abnormally, such as IL-6 and NOS2. STING plays the main role in 8-OHG-induced macrophage activation and infiltration. Importantly, both overload of iron and Gpx4 reduction can induce ferroptosis and boost the release of 8-OHG, which leads to activation of STING pathway and infiltration of macrophages during Kras-driven pancreatic cancer (77). Based on this research, it is clear that modulation of ferroptosis is becoming a therapeutic potential in pancreatic cancer because macrophage reduction or inhibition of the 8-OHG-STING pathway decreases ferroptosis-mediated pancreatic carcinogenesis. Additional studies will be required to delineate the possible effects of the cGAS-STING pathway on other types of cell death in pancreatic tumors.

### STING Modulates Immune Responses

The cGAS-STING pathway is also implicated in immune surveillance of tumors because type I IFNs can increase the infiltration of T cells and NK cells (78, 79). However, immunosuppressive cytokines reduce the number of cytotoxic and helper T cells in pancreatic cancer (80). In pancreatic tumors, the NK cells are functionally impaired (81, 82). As mentioned earlier, pancreatic TAMs have a large population of TAMs with immunosuppressive activities. Type I IFNs have been found to reduce the production of TAMs and stimulate anti-tumor functions of macrophages (83). Hence STING pathway plays an important role in modulating the anti-tumor responses in pancreatic cells through enhancing the expression of type I IFNs.

## STING Plays an Important Role in Pancreatic Cancer Biotherapy

### Interaction Between STING Pathway and Metformin

Metformin is a basic anti-diabetes medication suggested to benefit different types of cancers, including pancreatic cancer, by modulating various pathways (84). On the other hand, it has been suggested that cytosolic sensing of DNA triggers the HER2–AKT1 axis, which STING promotes and modifies TBK1. HER2 (a kind of receptor tyrosine kinase) is crucial to mediate the suppression of cytosolic DNA sensing. HER2 lets tumor cells tolerate the anti-tumor immunity more effectively because it can reduce cellular death and senescence in cancer cells. It inhibits tumors from responding to the production of IFNs as well (85). Notably, HER2-AKT signaling regulates the STING pathway negatively. A piece of research demonstrated that metformin could increase the production of CD4^+^ and CD8^+^ T cells in the TME through decreasing AKT phosphorylation and enhancing the STING expression in pancreatic cancer (86). Therefore, metformin would have the ability to inhibit pancreatic cancer growth through promoting the STING/IRF3/IFN-β pathway and can be applied in combination with other types of immunotherapy.

### STING Agonists and Vaccines as Monotherapy or Combined With Other Treatments

The application of the cGAS-STING pathway in cancer therapies is complex because it varies in different cancer types, and there are still some challenges in utilization of STING, for example, it can activate the adaptive immune responses by type I IFN production, but it is difficult to control the local level of type I IFNs in tumor cells. However, it has been suggested that STING signaling can boost cancer immunotherapies *via* cancer vaccines. For instance, Luo et al. suggested that STING-dependent vaccines can inhibit tumor growth and make a long-term anti-tumor memory (87). Recently, many kinds of STING agonists have been introduced for anti-tumor activities. 5, 6-dimethylxanthenone-4-acetic acid (DMXAA) is one of the STING agonists and can modulate the immune system and result in anticancer responses. Still, it can promote the cGAS-STING pathway only in mice, which cannot be a functional treatment for cancer patients. Another example of cGAS-STING pathway agonists is cytosolic cyclic dinucleotides (CDNs) which can enhance the production of type I interferons through activating TBK1/IRF3, NF-κB, and STAT6 pathways (88). STING agonists can also be involved in cancer vaccines and activate the immune system against carcinogenesis. Many other kinds of STING agonists can be used for cancer treatment. Still, their ability to target human STING and induce anti-tumor responses in clinical trials makes them more efficient and reliable.

Clinical data reported that KRAS and MYC oncogene signaling causes the suppression of type I IFN responses in pancreatic cancer cases (89). STING ligands activate inflammatory responses, which help to improve the adaptive immune responses to antigens released by radiation therapy. In addition, the combination of radiation therapy and STING agonists controls local and distant tumors through developing T cell immunity in murine models of pancreatic cancer (90). STING agonists can promote the activation of cytotoxic T cells and the production of cytokines and inhibit pancreatic cancer progression, so it is clear that STING agonists modulate the immune microenvironment of pancreatic cancer (91). Jing, W et al. reported that chemokines attracted by C-X-C Motif Chemokine Receptor 3 (CXCR3), which are also dependent on IFN, can be produced through STING activation, and it means that CXCR3 has a crucial role in the anti-tumor activity of STING agonist treatment in pancreatic cancer (92). However, a recent study showed that using STING agonist alone only extends survival time and all the experimental cases still died from tumor progression. It has also been suggested that biopolymers containing the combination of STING agonists and specific modified chimeric antigen receptor (CAR) T cells promote immune responses against tumor cells and significantly improve the overall survival of pancreatic cancer and melanoma mouse models (93). Lorkowski et al. also reported that the delivery of STING agonist plus a Toll-like receptor 4 (TLR4) agonist through immunostimulatory nanoparticle (immuno-NP) could increase the local infiltration of IFNβ in pancreatic tumors. As a result, these immune-NPs are a potent way to enhance the innate immune responses against pancreatic cancer (94). Nowadays, the utilization of vaccines for cancer treatment has been a fascinating topic, and some vaccine-based studies have been administrated in pancreatic cancer as well (95). Kinkead et al. used a different kind of vaccine based on STING in murine pancreatic cancer models. This vaccine targets neoantigens that are arisen from somatic mutations, and its integration with checkpoint modulators (anti–PD-1 and agonist OX40 antibodies) increases anticancer immune responses (96). Based on this body of research, different kinds of STING agonists have been applied to treat pancreatic cancer, shown in **Table 1**.

**Table 1:** Involved STING agonists in pancreatic cancer treatment.

STINGagonists	Used method during studies	Type of cancer cells	Results	References
DMXAA	Combined with chemotherapy (Gemcitabine)	KPC mouse model of pancreatic cancer	Increased the survival rate and anticancer immunity through prompting T cells	(91)
DMXAA	Monotherapy	KPC mouse model of pancreatic cancer	Reduced tumor size by activating cytolytic T cells	(91)
3′3′-cGAMP	Loaded in engineering polylactic-co-glycolic acid (PLGA) microparticles	KPC mouse model of pancreatic cancer	Decreased metastasis and tumor growth and promoted the anti-tumor immune responses	(97)
ADU-V19	Loaded in cancer vaccines and combined with checkpoint regulators	KPC mouse model of pancreatic cancer	Enhanced the vaccine immunogenicity, vaccine-specific T cells, and anticancer immune responses	(96)
ADU-S100	Monotherapy	KPC mouse model of pancreatic cancer	Stimulated immune responses by growing the expression of CXCR3 in T cells	(92)
cdGMP	Combined with TLR4 agonists	KPC mouse model of pancreatic cancer	Increased the immune cell population by activating APCs	(94)
cdGMP	Combined with chimeric antigen receptors (CAR T cells)	KPC mouse model of pancreatic cancer	Activated APCs initiated endogenous tumor-specific lymphocytes, and inhibited metastasis	(93)
IACS-8803	Combined with checkpoint blockade therapy	KPC mouse model of pancreatic cancer	Increased the population of lymphoid myeloid and enhanced the checkpoint blockade	(98)

## Conclusion

The genetic instability of cancer cells causes the presence of cytosolic DNA, which plays the main role in the activation of the cGAS-STING pathway. The cGAS-STING pathway has recently provided insights into its influences on cancer development. STING signaling performs an anti-tumor function in the tumor microenvironment through type I IFNs, associated with a better prognosis. Also, several studies have aimed to discover the exact role of STING in tumor immunity and treating multiple cancers. Pancreatic cancer, which has a special tumor microenvironment, has been studied to get more information about the effectiveness of immunotherapy for its treatment. Many pieces of research revealed that STING is an effective strategy for inducing the progression of pancreatic cancer and consequently anti-tumor activity, which has been recently applied to other therapies such as vaccines and immune-targeted nanoparticles. Although we have tried to sum up the potential roles of STING signaling in more detail in pancreatic cancer, it is still required to carry out deep studies to clarify different mechanisms in which STING can be activated and utilized for treatment purposes.

## Author Contributions

GM designed and drafted the manuscript. JL, AA, LD, Y-sW, and CW discussed and revised the manuscript. All authors contributed to the article and approved the submitted version.

## Funding

This work was supported by the National Natural Science Foundation of China (No.81874040, 82172350). This work was also supported by a grant from the Key Research and Developmental Program of Shandong Province (2018YFJH0505) and the Taishan Scholars Program (2019GSF108218).

## Conflict of Interest

The authors declare that the research was conducted in the absence of any commercial or financial relationships that could be construed as a potential conflict of interest.

## Publisher’s Note

All claims expressed in this article are solely those of the authors and do not necessarily represent those of their affiliated organizations, or those of the publisher, the editors and the reviewers. Any product that may be evaluated in this article, or claim that may be made by its manufacturer, is not guaranteed or endorsed by the publisher.

## Abbreviation

STING, Stimulator of interferon genes; TME, Tumor microenvironment; MDSC, Myeloid derived suppressor cells; TAM, Tumor-associated macrophages; DC, Dendritic cells; Treg, Regulatory T cell; TAM, Tumor-associated macrophage; APC, Antigen-presenting cells; IFN, Interferon; IFNAR, Interferon-α/β receptor; CAF, Cancer-associated fibroblasts; cGAMP, Cyclic guanosine monophosphate-adenosine monophosphate; cGAS, cyclic guanosine monophosphate-adenosine monophosphate synthase; IRF, Interferon regulatory factor; ER, Endoplasmic reticulum; PAMP, Pathogen-associated molecular pattern; PRR, Pattern recognition receptor; GTP, Guanosine 5′-triphosphate; ATP, Adenosine 5′-triphosphate; TBK1, Tank-binding kinase 1; STAT, Signal transducer and activator of transcription proteins; JAK, Janus kinase; ISGF, Interferon-stimulated gene factor; MLKL, mixed-lineage kinase domain-like protein; TNFα, Tumor necrosis factor alpha; MFN, mitofusins; GPX4, Glutathione peroxidase 4; 8-OHG, 8-Oxyguanine; NOS2, Nitric oxide synthase HER2, Human epidermal growth factor receptor 2; DMXAA, 5, 6-dimethylxanthenone-4-acetic acid; CDN, Cyclic dinucleotide; NF-κB, Nuclear factor-kappa B; CXCR3, C-X-C motif chemokine receptor 3; CAR T cells, chimeric antigen receptor T cells; TLR4, Toll-like receptor 4.
